# Implications of glial nitric oxide in neurodegenerative diseases

**DOI:** 10.3389/fncel.2015.00322

**Published:** 2015-08-17

**Authors:** Jose Enrique Yuste, Ernesto Tarragon, Carmen María Campuzano, Francisco Ros-Bernal

**Affiliations:** ^1^Neurobiotechnology Group, Departament of Medicine, Facultat de Ciències de la Salut, Universitat Jaume ICastelló de la Plana, Spain; ^2^Département des Sciences Biomédicales et Précliniques/Biochimie et Physiologie du Système Nerveux, Centre de Recherche du Cyclotron, Université de LiègeLiège, Belgium

**Keywords:** nitric oxide, neuroinflammation, neurodegenerative disorders, neuronal nitric oxide, neuronal death

## Abstract

Nitric oxide (NO) is a pleiotropic janus-faced molecule synthesized by nitric oxide synthases (NOS) which plays a critical role in a number of physiological and pathological processes in humans. The physiological roles of NO depend on its local concentrations, as well as its availability and the nature of downstream target molecules. Its double-edged sword action has been linked to neurodegenerative disorders. Excessive NO production, as the evoked by inflammatory signals, has been identified as one of the major causative reasons for the pathogenesis of several neurodegenerative diseases. Moreover, excessive NO synthesis under neuroinflammation leads to the formation of reactive nitrogen species and neuronal cell death. There is an intimate relation between microglial activation, NO and neuroinflammation in the human brain. The role of NO in neuroinflammation has been defined in animal models where this neurotransmitter can modulate the inflammatory process acting on key regulatory pathways, such as those associated with excitotoxicity processes induced by glutamate accumulation and microglial activation. Activated glia express inducible NOS and produce NO that triggers calcium mobilization from the endoplasmic reticulum, activating the release of vesicular glutamate from astroglial cells resulting in neuronal death. This change in microglia potentially contributes to the increased age-associated susceptibility and neurodegeneration. In the current review, information is provided about the role of NO, glial activation and age-related processes in the central nervous system (CNS) that may be helpful in the isolation of new therapeutic targets for aging and neurodegenerative diseases.

## Introduction

Nitric oxide (NO) was discovered as an endothelium-derived relaxing factor more than two decades ago, and since then, its participation in a widening number of pathways has been continuously reported. There is increasing evidence showing that alterations in the NO signaling may be related with different diseases as it plays a key role in diverse neurodegenerative-associated processes such as neuronal death, necrosis, apoptosis and autophagy (Calabrese et al., 2007). In particular, it has been suggested that S-nitrosylation is involved in the pathogenesis of various neurodegenerative disorders including Parkinson’s disease (PD), amyotrophic lateral sclerosis (ALS), multiple sclerosis (MS) and Alzheimer’s disease (AD). The neuroinflammation that characterize these pathologies is largely associated with the production of NO; it is the aim of this review to describe how these NO-induced outcomes are produced, as well as trying to explain why they are important in the context of neurodegeneration. Further understanding of how imbalanced NO metabolism can contribute to neuronal cell death is determinant to formulate achievable strategies for the prevention and treatment of neurodegenerative disorders. Moreover, as NO acts as a double-edged sword contributing both positively and negatively, or even simultaneously, to these diseases it is important to disentangle the effects of this molecule in order to attempt rational interventions towards them.

## NO Signaling Pathways

In mammals, NO is mainly synthesized by nitric oxide synthases (NOS) through the conversion of L-arginine to NO and L-citrulline (Knowles and Moncada, 1994). Traditionally, three isoforms of NOS have been identified in central nervous system (CNS): NOS1 or neuronal NOS (nNOS), NOS2 or inducible NOS (iNOS) and NOS3 or endothelial NOS (eNOS; Alderton et al., 2001). These three isoforms differs in their activity patterns: (i) nNOS localizes to synaptic spines, astrocytes and the loose connective tissue surrounding blood vessels in the brain; (ii) iNOS is a calcium (Ca^2+^)-independent isoform not constitutively expressed by astrocytes and microglia but these glial cells often expressed this isoform in pathological conditions such in response to inflammatory stimuli (Saha and Pahan, 2006); and (iii) eNOS is present in both cerebral vascular endothelial cells and in motor neurons (Estévez et al., 1998). The activity of iNOS is tightly associated with its expression levels and is induced during cell inflammatory response while nNOS and eNOS activities depend on intracellular Ca^2+^ levels and their CNS expression.

**Figure 1:**
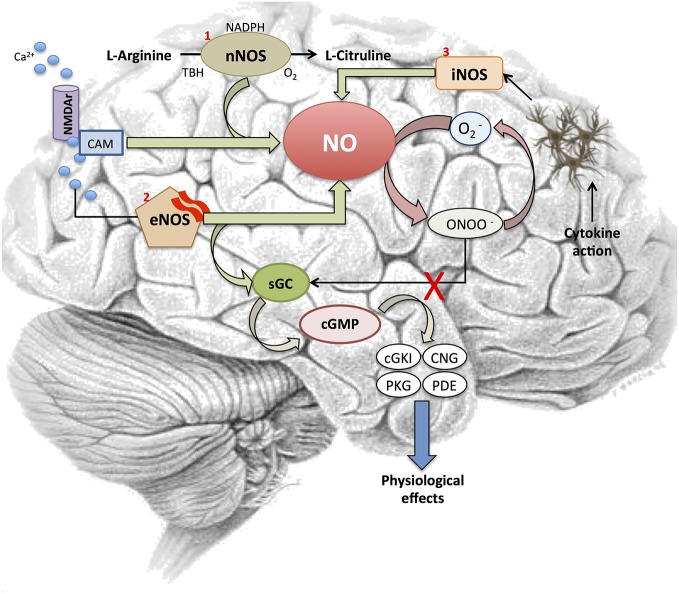
**Nitric oxide signaling pathway.** Figure shows different steps in the NO signaling cascade and its interaction with several elements involved in the signaling pathway. NO is synthetized by two Ca^2+^-dependent or one independent Ca^2+^-mediated processes. First, (1) NOS1 or neuronal NOS (nNOS)-catalyzed reaction converts L-arginine into L-citrulline in the presence of O_2_, nicotinamide adenine dinucleotide phosphate (NADPH) and tertiary-butyl hydroperoxide (TBH) after the activation of the NMDA receptor by Ca^2+^. Also, (2) intracellular Ca^2+^ activates eNOS to release NO from brain microvessels. This NO binds to soluble guanylyl cylclase (sGC) receptors, which trigger a cGMP-dependent pathway and interacts with its downstream effectors (cGKI, CNG, PKG, PDE), the ultimate mediators of the NO’s physiological response. In addition, sGC is also critically affected by redox status. NO initiates the synthesis of ONOO^−^ when O_2_^−^ is present, which results in a dysfunctional uncouple variety of nitric oxide synthases (NOS) that produces O_2_^−^ rather than NO. Finally, (3) NO is synthesized following the transcriptional expression of a Ca^2+^-independent iNOS isoform in glial cells, astrocytes and microglia after cytokine exposure.

To date, soluble guanylyl cyclase (sGC) is the most accepted physiological receptor described for NO. This receptor is formed by α and β subunits together with a prosthetic heme group with a ferrous iron. The binding of NO to this receptor activates the C-terminal catalytic domain, which produces guanosine 3′,5′-cyclic monophosphate (cGMP; Stamler et al., 1997). This enzyme activity is critically affected by redox status as the oxidation of the heme moiety on the β-subunit turns the enzyme sensitive to NO. There are other mechanisms by which oxidative stress may compromise this cGMP synthetic pathway (Figure 1). For instance, reactive oxidant peroxynitrite (ONOO^−^) induced by NO in the presence of superoxide (O2^−^) results in a dysfunctional uncouple variety of NOS that produces O2^−^ rather than NO under oxidative stress (Xia et al., 1998; Sasaki et al., 2008).

In response to NO, sGC activity increases more than 200 fold. The increased level of cGMP activates selected pathways to induce cellular responses (Stamler et al., 2001). Briefly, the cGMP produced by NO-activated sGC directly interacts with its downstream effectors, such as cGMP dependent kinase (cGKI or PKG), cyclic nucleotide gated (CNG) channels and cGMP dependent phosphodiesterase (PDEs). There is great scientific agreement about the nNOS and eNOS implication in NO production in skeletal and cardiac muscle (Kobzik et al., 1994; Sartoretto et al., 2011). This endogenously produced NO can promote two physiological functions differentiated by its cGMP-dependence: (i) to induce relaxation through the cGMP signaling pathway (Balligand et al., 1993; Mohan et al., 1996); and (ii) to modulate increases in contraction independent of cGMP concentration (Kobzik et al., 1994). The activation of such elements is the preferred mechanism by which NO mediates most of its physiological effects including vascular smooth muscle tone and motility, phototransduction and maintaining fluid and electrolyte homeostasis (Palmer et al., 1987; Bredt et al., 1990; Knowles and Moncada, 1994).

However, emerging evidence suggests the participation of NO in another signaling mechanism: “S-nitrosylation” of target proteins. S-nitrosylation is a non-enzymatic post-translational modification consisting in a covalent addition of a NO group to a cysteine thiol/sulfhydryl (RSH). This S-nitrosylation participates in a huge number of physiological events including those implicated in muscular contraction (Xu et al., 1998), cellular trafficking (Ozawa et al., 2008), circulation (Singel and Stamler, 2005) and apoptotic pathways (Benhar et al., 2008; Cho et al., 2009). Coherent with this, a main implication of ryanodine receptor 1 (RyR1) has been found in the activation and S-nitrosylation of Ca^2+^ release channel in sarcoplasmic reticulum of skeletal muscle by low concentration of NO (Eu et al., 2000).

Based on these studies, a possible involvement of S-nitrosylation in neuronal function has been suggested in the brain, particularly in the cerebellar Purkinje cell layer and dentate gyrus where RyR1 messenger RNA (mRNA) is mostly prominent (Mori et al., 2000). However, more studies have to be performed to corroborate a preponderant S-nitrosylation pathway in CNS and its implication in neuroinflammatory processes and neurological disorders.

## Neuroinflammation and Nitric Oxide

Neuroinflammation represents the coordinated cellular response to tissue damage and is characterized by the microglial release of pro-inflammatory factors such as cytokines, proteases and toxic free radicals. The progress associated with neuroinflammation can be acute or chronic, while the appropriate regulation of this general process facilitates recovery, uncontrolled neuroinflammation can induce a secondary injury. The main purpose of acute neuroinflammation is to remove the source of harm in order to restore the brain to a healthy condition. However, a maintained response is known to induce neuronal dysfunction and death (McGeer et al., 2003).

Neuroinflammation has been demonstrated to be closely associated with the pathogenesis of several psychiatric illnesses and neurodegenerative diseases like AD, PD and Huntington’s disease (Bales et al., 2000; Hunot and Hirsch, 2003; Doorduin et al., 2009; Silvestroni et al., 2009; Dobos et al., 2010; Rao et al., 2010). Moreover, there is evidence showing that this condition is detectable years before significant loss of neurons occurs (Frank-Cannon et al., 2009; Fuhrmann et al., 2010; Ratai et al., 2011), hence its relevance in the context of neurodegenerative disorders. This paradigm is supported by several studies showing that a long-term treatment with Non-steroidal anti-inflammatory drugs (NSAIDs) may have a preventative effect in neurodegenerative diseases as the above mentioned (McGeer and McGeer, 2007; Wahner et al., 2007).

**Figure 2:**
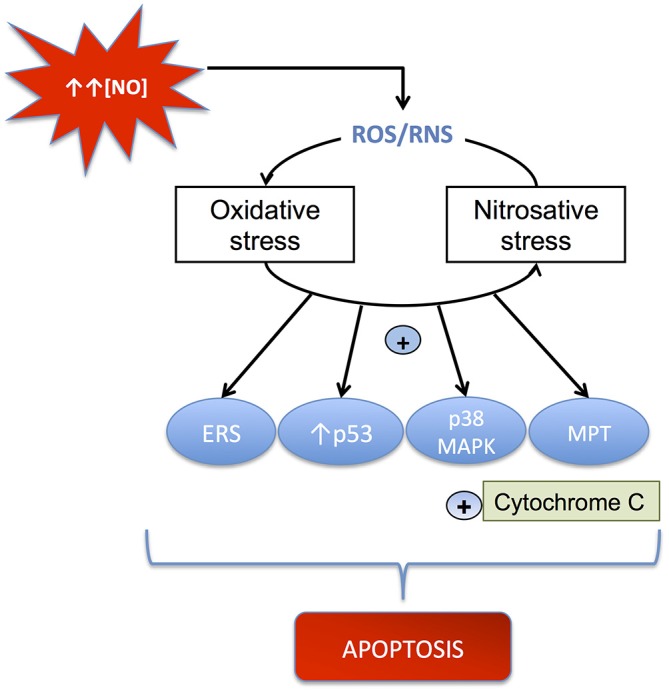
**Mechanisms through which ROS/RNS cause apoptosis.** NO can induce oxidative and nitrosative stress, which activates mitochondrial apoptosis by several pathways, including: (i) stimulation of mitochondrial permeability transition (MPT); (ii) up-regulation of p53; (iii) activation of the p38 MAP kinase pathway; and (iv) induction of endoplasmic reticulum stress (ERS). All these processes produce cytochrome c release and apoptosis.

Neuroinflammation-induced cell death is often derived from the long-term impact caused by the increase of reactive oxygen and nitrogen species (RONS), which play a major role in eliciting apoptotic cell death through irreversible oxidative or nitrosative injury to neuronal elements (Nakagawa and Yokozawa, 2002). The brain is highly susceptible to oxidative stress due to its imbalance between an efficient antioxidant defense system and its capacity to generate oxidative species. As a matter of fact, the brain presents low levels of glutathione (GSH) and moderate activity of the antioxidant enzymes catalase, superoxide dismutases (SODs) and GSH peroxidase. On the contrary, the elevated levels of ascorbic acid, the high concentration of transition metals such as copper and iron, and the huge aerobic metabolism contribute to the generation of oxidative (ROS/RNS) species eliciting necrotic neuronal death (Figure 2).

There is an intimate relation between glial activation and neuroinflammation in the human brain. The presence of activated microglia was initially considered as a sensitive marker to identify potential sites of tissue destruction (Mosley et al., 2006; Galea et al., 2007). Even more, the engagement of astrocytes and endothelial cells, the observed activation of microglia and its implication in neuroinflammatory processes, have been widely demonstrated to derive from the affection of neuronal viability through a persistent RONS generation.

The role of NO in neuroinflammation has been determined in animal models associated with excitotoxicity processes induced by glutamate accumulation and microglial activation. Released NO from activated microglia acts at the presynaptic site blocking the reuptake of glutamate, thus inducing the activation of N-Methyl-D-aspartate (NMDA) receptors and facilitating neuronal death (Rao et al., 2007, 2012; Kim et al., 2009). The regulation of iNOS isoform, highly implicated in neuroinflammatory processes associated with glial cells, takes place at the transcriptional level. Several transcription factors are implicated in trans-activation of iNOS gene, among them the nuclear factor *k*-light-chain-enhancer of activated B cells (NF-κB) is the most important one. Without the inhibition from the NF-κB p50:p65 arresting protein (IκB) NF-κB translocates to the nucleus and binds κB elements in iNOS promoter (Davis et al., 2005; Kanarek et al., 2010). In glial cells, this NF-κB-mediated iNOS expression triggers several pathways related with RONS formation, caspase and nNOS signaling activity and mevalonate production. It has been demonstrated that chronic NMDA administration up-regulates the levels of proinflammatory IL-1β, TNF-α, glial fibrillary acidic protein (GFAP) and iNOS in rat brains (Chang et al., 2008). Altogether these findings suggest that there is cross-talk between neuroinflammation and excitotoxicity that involves NO release and iNOS up-regulation in the brain.

However, it seems clear that NO modulation of inflammatory processes requires other mechanisms besides microglial cytokine release. The interaction with key regulatory pathways might be one of these mechanisms. Coherently, it is known that NO inhibits NF-κB activation thus controlling inflammation both in muscular cell lines (Hattori et al., 2004) and through release by nitrooxyphenyl acetylsalicylate (NO-ASA), a non-steroidal anti-inflammatory drug (NO-NSAID) in cancer cell lines (Kashfi and Rigas, 2005). Moreover, S-nitrosylation of NF-κB protein was the mechanistic role for NO-action resulting in diminished binding of this protein to DNA for transcriptional activation (Chattopadhyay et al., 2010).

Historically, astrocytes function in CNS injuries was reduced to maintain ionic homeostasis and participate in glial scar formation and tissue repair, which limits inflammation and promotes tissue repair, secreting nerve growth factors (Simard and Nedergaard, 2004). However, since NO and IL-1β are also produced by activated astrocytes, recent studies support an important active role of these cells in neuroinflammation and the neurodegeneration associated with dysregulations in NF-κB pathway and in NO production. Both dysregulations associates with a crosstalk between lipid mediators, such as sphingosin, and signaling inflammatory cytokines (Spiegel and Milstien, 2011).

## Nitric Oxide and Neurodegenerative Diseases

Selective neuronal death is typical of most neurodegenerative diseases including PD, AD, ALS and MS (Guix et al., 2005). The participation of oxidative stress in the development of several neurodegenerative disorders has been largely documented (Calabrese et al., 2007), with NO suggested as a starring character (Chabrier et al., 1999). This relationship was evidenced by the fact that increased nitration of protein aggregates was prominent in different synucleinopathies and tauopathies (Duda et al., 2000; Horiguchi et al., 2003).

The augmented nitration of proteins can be initiated by an increase in the production of NO during neuroinflammation and the generation of free radicals by dysfunctional mitochondria, which are commonly observed in various neurodegenerative disorders (Guix et al., 2005; Pacher et al., 2007). Moreover, it has been demonstrated that NO is able to activate molecular elements, such as cyclooxygenase (COX; Mollace et al., 2005), which is typically up-regulated in brain cells under inflammatory conditions (Mancuso et al., 2007). In addition, the combination of NO and free radicals like the superoxide anion will result in the formation of highly reactive peroxynitrite. Peroxynitrite can then nitrate tyrosine residues on proteins to 3-nitrotyrosine, induce lipid peroxidation, and cause DNA damage (Ischiropoulos and Beckman, 2003).

NO is especially harmful under pathological conditions involving the production of RONS (Wahner et al., 2007) and ONOO^–^ formation. Nitrotyrosination inhibits tyrosine phosphorylation and hence affects the signal transduction pathways of growth factor (Jonnala and Buccafusco, 2001). Moreover, the presence of nitrotyrosination has been described in several neurodegenerative diseases linked to oxidative stress, such as AD (Guix et al., 2012), PD (Good et al., 1998) and ALS (Cookson and Shaw, 1999; Smith and Lassmann, 2002).

In sum, diverse stimuli ranging from neuronal impaired pathway-associated products to environmental toxins can trigger glial dysregulations. In neurodegenerative diseases, alterations derived from overactivated glia, microglia and astroglia are particularly present.

### Alzheimer’s Disease

The accumulation of β amyloid (Aβ) plaques and neurofibrillary tangles are the histopathological gold-standard hallmark for AD diagnosis. Together with these, the contribution of neuroinflammatory processes to the aging brain and the development of Alzheimer’s and other neurodegenerative diseases is also well documented (Zhang et al., 2013; Mosher and Wyss-Coray, 2014). However, the exact mechanism by which microglial activation is disturbed in AD is still not completely understood.

Accordingly, a critical role of NO in the development of AD has been suggested, as neuronal cell loss, neuronal injury and protein misfolding are reported to occur as a consequence of NO overproduction (Nunomura et al., 2001; Nakamura and Lipton, 2011; Swerdlow, 2011). Significantly, nitrated form of protein tau has been reported in NFTs and neuritic plaques in brains of AD patients as well (Reynolds et al., 2006).

There is evidence that link NO production with mitochondrial dysfunction and neuroinflammation, especially as regards glial response (Jekabsone et al., 2007). Moreover the pro-inflammatory and toxic effects of amyloid in neurons co-cultured with glia are hampered by iNOS inhibitors (Brown, 2007).

Furthermore, it has been suggested that microglial nicotinamide adenine dinucleotide phosphate (NAPDH) oxidase complex is the major source of ROS in the brain (Wilkinson and Landreth, 2006).

**Figure 3:**
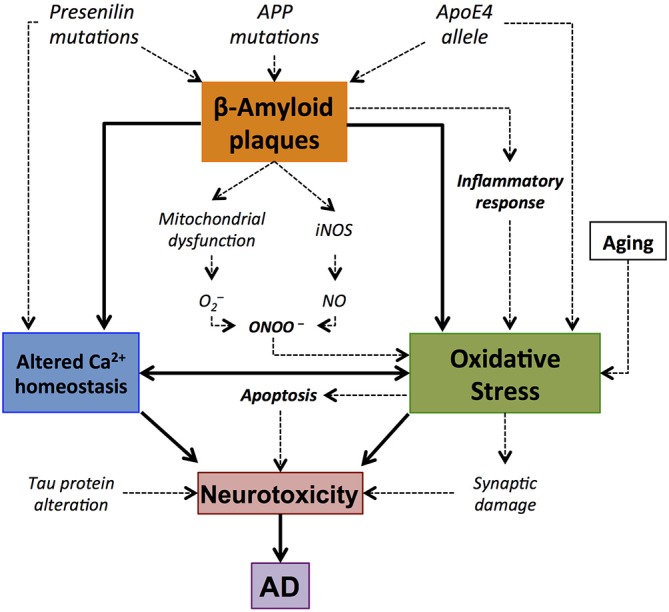
**Central role of amyloid β-peptide (Aβ) in the oxidative stress elements involved in Alzheimer’s disease (AD).** Accumulation of Aβ plaques results in oxidative stress. This oxidative stress might result from the implication of different pathways, such as mithocondrial dysfunction or inflammatory response, and it is manifested by synaptic damage and alterations in Ca^2+^ homeostasis. This may lead to apoptotic processes that result in the death of the cell and neurotoxicity. This is consistent with the concept of Aβ-associated oxidative stress and neurodegeneration in AD brain.

The imbalance produced by the detoxification of ROS prompts an increase in oxidative stress that has proved to be involved in several excitotoxicity processes (Ferrer et al., 2010). S-nitrosylation has also been implicated in AD (Lipton et al., 1993), exhibiting a modulatory effect on glutamatergic NMDA receptors (Lipton, 2007b). Over-stimulation of NMDA receptors may produce an excessive Ca^2+^ influx that can generate ROS and activate excitotoxicity processes that lead to cell death. Moreover, this excitotoxicity that has been suggested as a mediator of neurotoxicity in this neurodegenerative disorder (Lipton, 2007a), and specifically in neurons, may also activate nNOS and induce NO overproduction (Gu et al., 2010; Figure 3).

Studies in cell cultures suggest that glutamate-induced cell death after NO release and ROS results from the inhibition of mitochondrial respiration caused by glial activation (Beckman et al., 1994; Loihl and Murphy, 1998; Bal-Price and Brown, 2001). Interestingly, Aβ stimulates the production of NO, which turns to be also a proinflammatory marker of microglial activation. There is evidence of an interaction between Aβ aggregates and microglia, as it was demonstrated that the later binds to the former through membrane receptors such as TLR2, 4, 6, and 9 (Bamberger et al., 2003), and this has been suggested as a constituent of the inflammatory process in this disease. In addition, there is evidence of Nod-like receptor (NLR) family, pyrin domain containing 3 (NLRP3), activation in a mouse model of AD (Lambert et al., 2009). Interestingly, NLRP3 is a modulator of IL-1β, a proinflammatory cytokine which production is also stimulated by the presence of Aβ plaques, together with other inflammatory markers, including IL-6 and tumor necrosis factor alpha (TNF-α; Jekabsone et al., 2006; Jimenez et al., 2008).

The description of the mechanism by which Aβ increases the production of NO is still incomplete. One explanation is that progressive Aβ accumulation disrupts the Ca^2+^ homeostasis causing the before mentioned rises in NO (Cetin et al., 2013). Also, an increase of lipid peroxidation in the cellular membrane has been observed in the presence of Aβ accumulation (Xie et al., 2010). Another source of Aβ-induced increase in oxidative stress is the known interaction between both APP and amyloid plaques with mitochondrial proteins, which leads to alterations in normal function (Spuch et al., 2012).

Nitrosative stress has also been associated with pathological alterations (Nakamura and Lipton, 2011) since this process increases the aggregation of Aβ in early stages of AD, impairing the formation of hippocampal long-term potentiation (LTP; Kummer et al., 2011; Thiabaud et al., 2013). In addition, increasing oxidative stress in cultured hippocampal neurons led to nitrotyrosination of presenilin protein (PSEN1), which finally induced an enhancement of total Aβ (Guix et al., 2012). Interestingly, AD brains show similar increased levels of nitrotyrosinated PSEN1 in comparison with age-matched controls (Guix et al., 2012), which remarks the relevance of oxidative stress to the neuroinflammatory process and the progress of pathophysiological hallmarks in this disease.

### Parkinson’s Disease

PD is an age-related neurodegenerative disease characterized by a prominent loss of dopaminergic neurons in the substantia nigra (SN; Dauer and Przedborski, 2003; Danielson and Andersen, 2008). The loss of DA modulation triggers a complex series of neurochemical, anatomical, and electrophysiological alterations that lead to persistent changes in striatal neurons and their signaling pathways (Wang and Pickel, 2002; Bamford et al., 2004; Picconi et al., 2004). Significant evidence indicates that chronic inflammatory response, mainly triggered by activated microglia and astroglia has a crucial role among the pathogenic mechanisms contributive to degeneration of dopaminergic neurons (McGeer et al., 1988, 2003; Barcia et al., 2004; Benner et al., 2008).

As in AD, the NO-induced glial activation has also a detrimental effect related to PD. The pathophysiology of microglial activation due to increases in oxidative stress causes an increased uptake of manganese inside the cell, which has been linked to the density of microglial cells especially in the basal ganglia (Gonzalez-Cuyar et al., 2014).

Substantial evidence demonstrates the involvement of NO in the degeneration of dopaminergic neurons in the SNpc (Jenner, 2003) and along the nigrostriatal pathway (Duncan and Heales, 2005; Zhang et al., 2006). It has also been demonstrated that nitrotyrosination can inhibit tyrosine hydroxylase (Kuhn and Geddes, 2002), and it is known that monoamine oxidase B (MAO-B) generates H_2_O_2_ during the catecholamine metabolism (Tipton, 1967). This is interesting because the activity of MAO-B, which is located in the mitochondrial membrane, is increased in aged population (Bhaskaran and Radha, 1983). This source of increased oxidative stress has been suggested as a risk factor for the development of PD (Jenner, 2003).

Neuroinflammation can be induced by several factors, such as exposure to either infectious agents or toxicants. Compounds with such proinflammatory characteristics have been recognized from some time now as significant contributors to the pathogenesis of PD. This is coherent with studies showing that the inhibition of complex I of the mitochondrial electron transport by 1-methyl-4-phenyl-1,2,3,6-tetrahydropyridine (MPTP) can cause human parkinsonism, and increased nitrotyrosine in Lewy bodies and oxidative damage (Beal, 1998, 2002). Moreover, rotenone, a widely used pesticide, has proved to cause a syndrome in rats that mimics typical pathology displayed by PD patients (He et al., 2003), including microglial activation and the presence of proinflammatory markers in the brain (Li et al., 2012).

**Figure 4:**
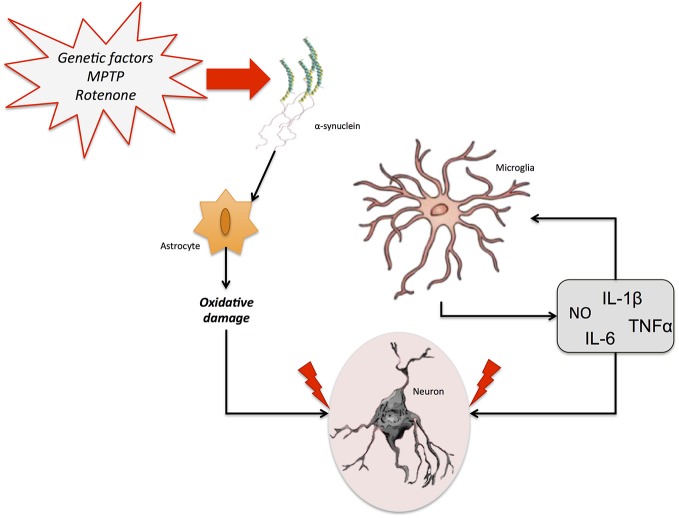
**Neuroinflammation in Parkinson’s disease (PD).** Different genetic and/or environmental factors such as parkin mutations or MTPT exposure lead to the accumulation of α-synuclein aggregates in the brain. This accumulation triggers the activation of glial cells. The proinflammatory cytokines freed by astrocytes and microglia stimulate the release of several neuroinflammatory markers, including NO, IL-6, IL-1β, and TNF-α, which can promote neuronal death and aggravate the neurodegenerative process.

A potential role of NO and NOS isoforms in the pathophysiology of PD has been emphasized. Increases in iNOS expression and NO-mediated modulation of the mitochondrial apoptotic pathway have also been observed after injection of lipopolysaccharide (LPS) or 6-OHDA in the SN and striatum in different experimental models of PD (Singh et al., 2005). It is worth mentioning that nNOS overexpression and the formation of peroxynitrite in polymorphonuclear leukocytes have been reported in PD patients (Gatto et al., 2000; Gilgun-Sherki et al., 2001). Interestingly, this peroxynitrite exposure has been also linked to the formation of α-synuclein aggregates (Souza et al., 2000). This is important, given that nitrated α-synuclein seems to contribute to the increased ROS production, decreased adenosine triphosphate (ATP) production, and degeneration of dopaminergic neurons, as well as to microglial activation, a reduction in the number of T-cells and increased cell death (Murray et al., 2003; Guix et al., 2005; Figure 4).

However, regardless all these data, the exact contribution of NO-dependent mechanisms to neurodegeneration and neuroinflammation in PD is still not completely understood. One of the proposed ideas is the production of ROS during the normal metabolism of dopamine (DA). In the human SNpc, the oxidation product of DA may polymerize to form neuromelanin, which has proved effective in aggravating the degenerative process through neuroinflammation (Zecca et al., 2008). On the other hand, although data from preclinical and clinical studies suggest that neuroinflammation could be a hallmark of the progression of the disease from an early asymptomatic stage (Lee et al., 2009). An elevated level of inflammatory cytokines, such as TNF-α and IL-6 NO-associated (Wilms et al., 2007) was found in the *post mortem* brain of PD patients. Importantly, an up-regulation of the genes encoding for these inflammatory cytokines, COX-2 and iNOS was observed in microglial cells from PD patients (Knott et al., 2000; Saha and Pahan, 2006). In sum, although later studies have shed light on the etiopathology and neuroinflammatory processes associated with PD, more studies are have to be developed since the exact mechanism through which neuroinflammation and NO-associated pathways contribute to the development or progress of PD remains elusive.

Interestingly, the S-nitrosylation of parkin, another element significantly implicated in familial form of PD, has shown to interfere with the protective properties of this protein. Briefly, parkin acts as a transcriptional repressor of p53 (da Costa et al., 2009). What various studies demonstrate is that the presence of oxidative stress in the form of S-nitrosylation diminishes parkin’s protective features (Chung et al., 2004; Sunico et al., 2013). Concretely, the addition of ubiquitin on specific substrates that parkin provides is impaired in the presence of NO. This evidence suggests that S-nitrosylation is directly implicated in the pathophysiology of PD by impairing the protective role of parkin.

### Multiple Sclerosis

**Figure 5:**
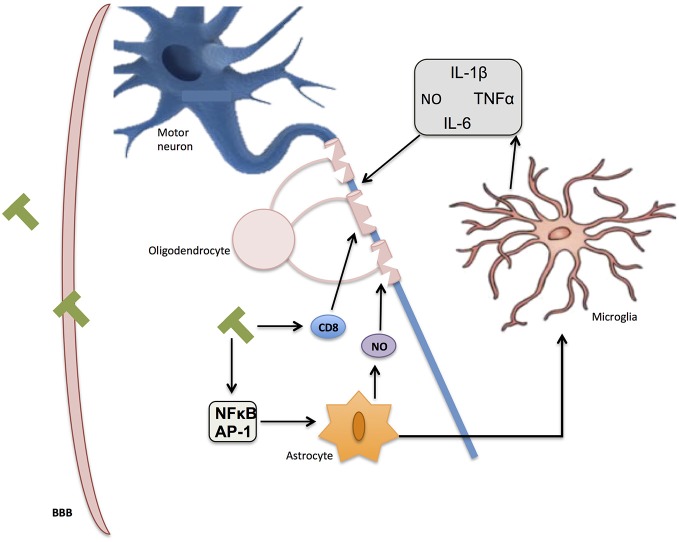
**Inflammation in multiple sclerosis (MS).** Immune T-cells bypassing the blood-brain barrier (BBB) affect oligodendrocyte structure and activate glial response through NF-κB and AP-1. Reactive oxygen species (ROS) and nitric oxide (NO) secretion by activated microglia and astrocytes further contribute to myelin damage, axon degradation, and ultimate neuronal death.

MS is an inflammatory disease in which the insulating myelin of SNC is damaged (Duncan and Heales, 2005). Of unknown etiology, this disease is characterized by an infiltration of inflammatory mononuclear cells into the CNS through a damaged blood-brain barrier (BBB), which causes the release of inflammatory and cytotoxic mediators, including NO (Smith and Lassmann, 2002). This neuroinflammation elicits the infiltration of T lymphocytes, the recruitment of macrophages, astrocytic damage and the local activation of microglia (Gay et al., 1997; Nikić et al., 2011). Although there is a strong correlation between neuroinflammation and axonal damage, the exact mechanism of this damage has to be elucidated (Figure 5).

Importantly, since NO expression participates in the homeostatic maintenance of the BBB permeability, this molecule may have an essential role in MS. Additionally in this BBB breakdown different pathways might be involved inducing oligodendrocyte injury and loss of neuronal function (Smith and Lassmann, 2002). Although there is considerable evidence showing that the three NOS isoforms are involved in the pathophysiology of MS (Wu and Tsirka, 2009; AlFadhli et al., 2013), iNOS would play, together with cyclic guanosine monophosphate (Janigro et al., 1994; Hurst and Fritz, 1996; Mayhan, 1999) and the overproduction of RNS (Mayhan, 1999; Kean et al., 2000; Spitsin et al., 2000; Winkler et al., 2001), a particularly crucial role specially at the beginning of this disease (Duncan and Heales, 2005).

The first direct evidence of glial implication in this disease was the demonstration of an altered mitochondrial function, following the inhibition of microglial respiratory chain in an animal model of demyelination (Zielasek et al., 1995; Lu et al., 2000). In these studies, a tendency for impaired Nicotinamide adenine dinucleotide (NADH) dehydrogenase activity and a compensatory increase in cytochrome oxidase in chronic active MS plaques was also demonstrated. More recently, another role of microglia in MS has been clarified since the phagocytosis of neuronal debris, the result of neuronal and axonal damage, would contribute to ongoing neurodegeneration in this disease (Huizinga et al., 2012).

Initially, astrocytes have been assigned a secondary role in the lesion formation and repair in MS. However, recent literature has implicated astrocytes in both lesion development and repair depending on the lesion stage and topography (Brosnan et al., 1994). Additionally, increased iNOS and mRNA has been identified in astrocytes in *post mortem* studies (Bagasra et al., 1995), and the MS-associated characteristic of reversible axonal conduction blockade has also been demonstrated in *in vitro* conditions (Redford et al., 1997). However, iNOS reactivity in hypertrophic astrocytes has only been described in acute but not chronic MS lesions (Brosnan et al., 1994; Liu et al., 2001) associated with peroxynitrite overproduction. More recently, different studies have shown the contribution of brain-derived neurotrophic factor (BNDF)-dependent NO release (Colombo et al., 2012) and the NF-κB pathway associated with NO production and astrocytes activation to the regulation of cytokine and chemokine expression. Interestingly, both markers have been related to the regulation of the severity and progression of the disease (Brambilla et al., 2009).

Another crucial step for the recruitment of leukocytes to the CNS and evolution of MS pathology is the chemokine expression by microglia and macrophages. Associated with this expression and in response to interleukin release astrocytes are able to regulate the production of different chemokines such as C-X-C motif chemokine 12 (CXCL12; Calderon et al., 2006). Recently it was demonstrated that the excessive expression of iNOS is able to decrease the expression level of CXCL12 gene, which has been implicated in the restriction of immune cell invasion to the CNS and the neuroinflammation limit in animal model of MS (Petković et al., 2013). This suggests that down-regulating NO release and maintaining CXCL12 expression within the CNS could be a potential therapeutic approach to MS.

### Amyotrophic Lateral Sclerosis

ALS is mainly characterized by a progressive degeneration of motor neurons in the CNS that results in weakness, paralysis, and death (Long and Nguyen, 2013). The exact mechanism triggering this disorder is not totally understood, but within the primary hypotheses put forth to explain motor neuron degeneration, oxidative stress counts among the preferred theories (Rothstein, 2009).

There is evidence in mouse models of ALS that the administration of non-selective NOS inhibitors reduces motor neuron degeneration (Hyun et al., 2003). Accordingly, *post mortem* examinations of brains from patients with ALS show high levels of NO metabolites (Boll et al., 2003), together with clear protein and DNA damage caused by oxidation (Agar and Durham, 2003; Kato et al., 2005).

Another line of evidence proposes astroglial cells, specifically astrocytes, as the primary generator of NO-derived molecules contributing to both ALS initiation and development (Pehar et al., 2006; D’Amico et al., 2013). In this sense, some evidence demonstrates that astrocytes exposed to NO promote apoptosis of embryonic motor neurons (Cassina et al., 2002). Moreover, peroxynitrite has proved to also affect protein activity by oxidizing amino acid residues, as seen in nitrotyrosine, which is found in the CNS of both ALS patients (Abe et al., 1997; Bruijn et al., 1997) and ALS-mice models (Casoni et al., 2005; Yoshino and Kimura, 2006). Hence, peroxynitrite-mediated tyrosine nitration has been suggested as key for triggering neuronal degeneration in ALS (Beckman and Crow, 1993; Peluffo et al., 2004).

In addition, one of the genetic features found in more than 20% of ALS patients is an alteration in the gene encoding for the enzyme SOD-1, an intrinsic antioxidant (Reaume et al., 1996). Studies *in vitro* show that, differently from the augmentation in NO release found in normal SOD-1, mutant cells for this gene express fewer levels of NO (Cookson et al., 2002). This is coherent with the evidence indicating an association of this gene with the familial form of ALS (Conwit, 2006). Interestingly, the reaction of ONOO^–^ with the mutant form of SOD-1 has shown to have an effect on protein nitrotyrosination (Beckman and Crow, 1993), which is coherent with data obtained from clinical studies (Abe et al., 1995).

**Figure 6:**
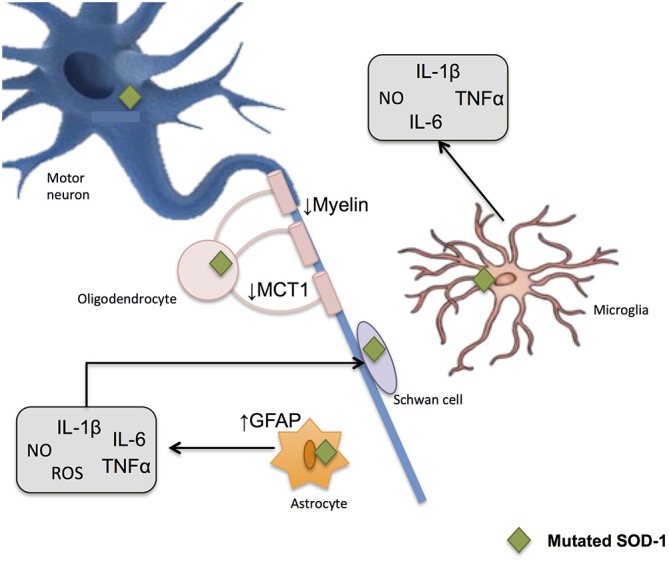
**Glial-induced neuroinflammation and neurotoxicity in amyotrophic lateral sclerosis (ALS).** Reactive astrocytes contribute to the degenerative process by influencing the activity of microglial and immune cells. An up-regulation of filament glial fibrillary acidic protein (GFAP) takes place and astrocytes increase the release of proinflammatory markers including NO and ROS. When mutated SOD1 accumulates within microglia, the later generates substances potentially harmful to other cells, thus potentiating neurotoxicity. Demyelinization and progressive loss of cholesterol is also observed after oligodendrocyte damage. These glial cells show a reduction in the monocarboxylate transporter 1 (MCT1), which in turn difficult the energy supplies to the neuron.

Together, all these data support the idea of a prominent role of oxidative harm as one of the principal cellular mechanisms of motor neuron degeneration (Beckman and Esteves, 2006; Figure 6).

## Conclusions and Perspectives

NO plays multiple roles in the nervous system and glial regulated pathways associated with neuroinflammation and neurodegenerative diseases. Under physiological conditions, it contributes to regulating proliferation, survival, and differentiation of neurons. It is involved in synaptic activity, neural plasticity, and cognitive function (i.e., memory); it also exerts long-lasting effects through regulation of transcription factors and modulation of gene expression. However, RNS generation also brings modifications to critical cysteine residues in proteins, including S­nitrosylation or nitrotyrosination.

If the physiological control of this signaling pathway fails, the pathological effects of NO and other RNS lead to (or are involved in) neuroinflammation and neurodegeneration processes. The NO-associated products resulted from the activation of glial response (either astrocyte or microglia) appear to especially contribute to the excitotoxic process that leads to neuronal death in several pathologies. More concretely, neurons appear particularly vulnerable to the effects of nitrosative stress. Susceptibility to NO and peroxynitrite exposure may depend on factors such as the intracellular antioxidants and stress resistance signaling pathways. Thus, NO redox signaling and modulation of the adaptive cellular stress responses, being released by glial cells or activating them, require further research to develop predictive means to deal with the increasing number of age-related neuropathological conditions.

Collectively, among NOS isoforms implicated in glial response related with neurodegenerative diseases, nNOS is the most implicated in a wide range of functions and pathologies with pleiotropic effects. In view of its ubiquitous expression in the CNS, there are extensive and unique chances for nNOS to interact with other neuronal elements, such as microglial and astroglial cells, thus exerting appropriate functional properties. Given increased nNOS activity and expression in many diseases, inhibiting nNOS might have putative therapeutic effects, among which anti-inflammatory properties can be hypothesized. Unfortunately, it will be a preferable means to interfere with specific pathway, for example, uncoupling nNOS–PSD95 interactions (Cao et al., 2005) since to date is impossible to inhibit nNOS directly without disturbing vital physiological functions and produce side effects.

## Conflict of Interest Statement

The authors declare that the research was conducted in the absence of any commercial or financial relationships that could be construed as a potential conflict of interest.

## Abbreviations

AD: Alzheimer’s disease
BBB: blood-brain barrier
cGMP: guanosine 3′,5′-cyclic monophosphate
CNS: central nervous system
DA: dopamine
eNOS: endothelial nitric oxide synthase
GSH: glutathione
GSNO: S-nitrosoglutathione
iNOS: inducible nitric oxide synthase
LPS: lipopolysaccharide
LTP: long term potentiation
MS: multiple sclerosis
NMDA: N-Methyl-D-aspartate
NO: nitric oxide
NOS: nitric oxide synthase
nNOS: neuronal nitric oxide synthase
NSAIDs: Non-steroidal anti-inflammatory drugs
PD: Parkinson’s disease
RONS: reactive oxygen and nitrogen species
sGC: soluble guanylyl cyclase.
